# Endoscopic endonasal retrieval of air gun pellet retained in the frontal sinus: A case report

**DOI:** 10.1016/j.ijscr.2022.107280

**Published:** 2022-06-06

**Authors:** Hassan Yousaf Shah, Rawan Emad Elshaer, Tarek Ziad Arabi, Belal Nedal Sabbah, Ghassan Alokby

**Affiliations:** a College of Medicine, Alfaisal University, Riyadh, Saudi Arabia; b Department of Otolaryngology-Head and Neck Surgery, King Faisal Specialist Hospital and Research Centre, Riyadh, Saudi Arabia

**Keywords:** ESS, endoscopic sinus surgery, OR, operating room, CSF, cerebrospinal fluid, Endoscopic sinus surgery, Air pellet, Foreign body, Frontal sinus

## Abstract

**Introduction and importance:**

Foreign bodies in paranasal sinuses are rarely encountered and most commonly present in the maxillary sinus. Guidelines for managing paranasal sinus object removal are limited due to its rarity. However, there are three major management options: open surgery, endoscopic sinus surgery, and observation.

**Case presentation:**

We report a rare case of an 18-year-old boy who underwent extended frontal sinus surgery to retrieve a retained air gun pellet in the right frontal sinus and repair the skull base defect resulting from the air gun pellet.

**Clinical discussion:**

Physicians commonly use endoscopic sinus surgery (ESS) for improving sinus drainage in recurrent chronic and acute infective sinusitis. Extended sinus surgery aims to maximize the communication between the paranasal sinuses and the nasal cavity. This extended communication helps improve access to the sinus, enhance drainage, and improve the delivery of topical medications. In addition, the use of ESS with the modified Lothrop procedure allows for better exposure of the skull base, which can help with the repair of a CSF leak.

**Conclusion:**

Based on our experience with this patient and similar literature, ESS should be considered a treatment option for patients with retained foreign objects in the frontal sinus.

## Highlights

• Conventional open surgeries are used to treat foreign body injuries; however, endoscopic approaches have become more feasible.
• Endoscopic sinus surgery allows for better visualization and reduced morbidity compared to open surgery.
• The modified Lothrop procedure should be considered a treatment option for patients with retained foreign objects in the frontal sinus.

## 1 Introduction

Foreign bodies in the paranasal sinuses are very uncommon, and roughly 70 % of instances are linked to maxillofacial trauma, while others are associated with dental procedures of the maxilla [1]. A study concluded that only about 2 % of penetrating head and neck trauma patients retained foreign objects in their paranasal sinus or skull base [2]. Sinus injuries from air guns account for a small percentage of head and neck penetrating traumas [2]. Air gun pellets do not commonly penetrate deep tissue because they have much lower kinetic energy than firearms [3].

Foreign bodies in the paranasal sinuses can be asymptomatic at the time of the initial presentation. They are often accidentally detected due to complications or a radiological workup for another cause; thus, they may go unnoticed unless they are directly sought out. As a result, even in relatively minor maxillofacial trauma cases, the presence of foreign bodies should be considered [4].

Reported complications of retained foreign body in the sinuses include chronic pain, sinusitis, abscess formation, and meningitis [5]. Management is directed by the patient's hemodynamic status, the site of the foreign body, and the degree of involved anatomy and essential structures such as the internal carotid, optic nerves, and ethmoidal arteries [6], [7], [8]. Early removal of foreign bodies is recommended to avoid serious complications [5], [9]. Historically, physicians treated these types of injuries by conventional open surgeries. However, due to advancements in imaging, endoscopic techniques can now be used to remove the object in endoscopic sinus surgery (ESS) [5].

ESS is a minimally invasive procedure primarily used to restore sinus ventilation and normal function [10]. Endoscopic surgery allows for better visualization and reduced morbidity compared to open surgery [11]. In this article, we present the case of an 18-year-old who underwent endoscopic sinus surgery (ESS) in an academic hospital after presenting with an air gun pellet in his frontal sinus. To the authors' knowledge, this is the second article reporting the usage of ESS to remove an air gun pellet in the frontal sinus [12]. This case report is reported in line with the SCARE criteria [13].

## 2 Case presentation

An 18-year-old male with a history of seizure disorders presented to the clinic with a right supraorbital air gun pellet wound. The patient did not have any other relevant medical, family, or drug use history. Initially, he was taken to the operating room (OR) for surgical exploration, but they could not remove the bullet. Therefore, he was referred to our clinic one-week post-injury with a history of vertical diplopia on downward gaze that developed after his injury. His gunshot wound was healing, dry, and clean.

**Fig. 1:**
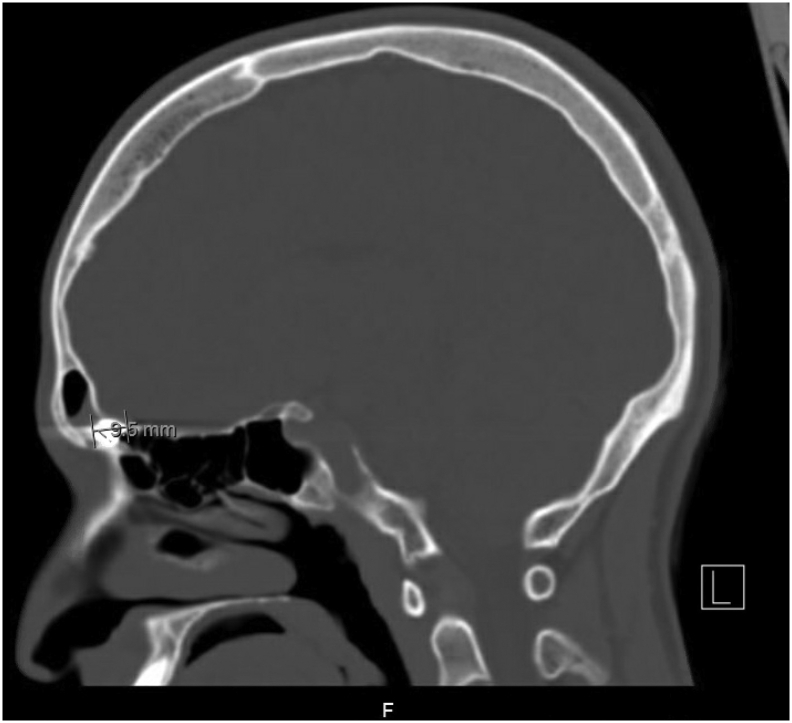
CT showing a foreign object in the frontal sinus. Parasagittal CT of the paranasal sinuses on bone window showing 9.5 mm wide foreign object (air pellet) in the inferior aspect of the frontal sinus.

**Fig. 2:**
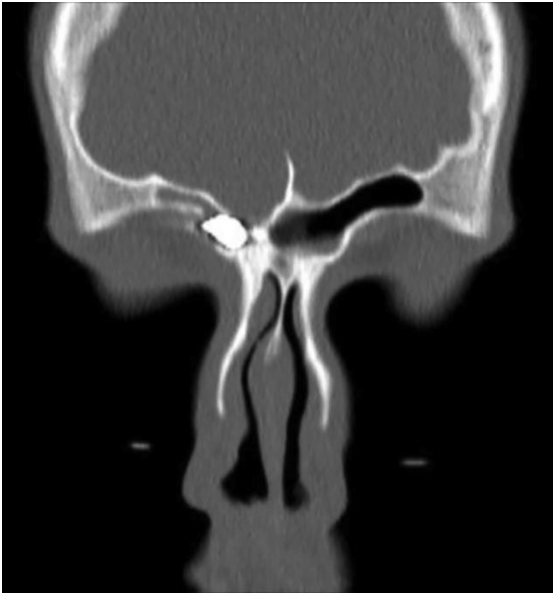
CT revealing a fractured posterior table of the frontal sinus. Coronal CT of the paranasal sinuses on bone window showing a fractured posterior table of the frontal sinus.

CT scan (Fig. 1) showed a metallic foreign body located in the inferior aspect of the right frontal sinus and measuring up to 12 mm in diameter. Moreover, there is a fracture of the adjacent superomedial wall of the right orbit and the posterior table of the frontal sinus (Fig. 2). The brain parenchyma was intact, and no contusions or hemorrhages were found. After patient counseling, he was booked for endoscopic sinus surgery to remove the foreign body.

**Fig. 3:**
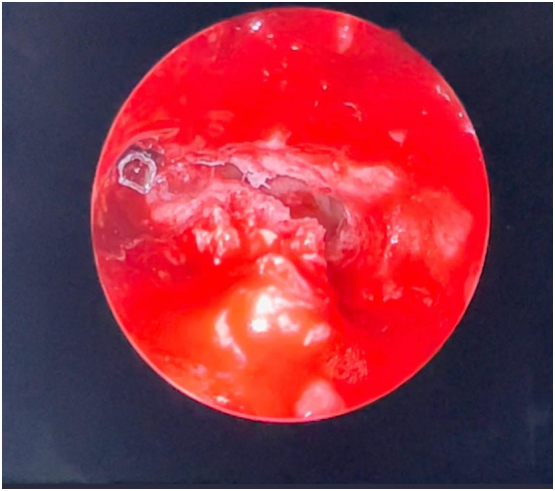
Endoscope showing retained bullet. Endoscopic endonasal view of the frontal sinus showing the bullet after performing the modified Lothrop procedure.

**Fig. 4:**
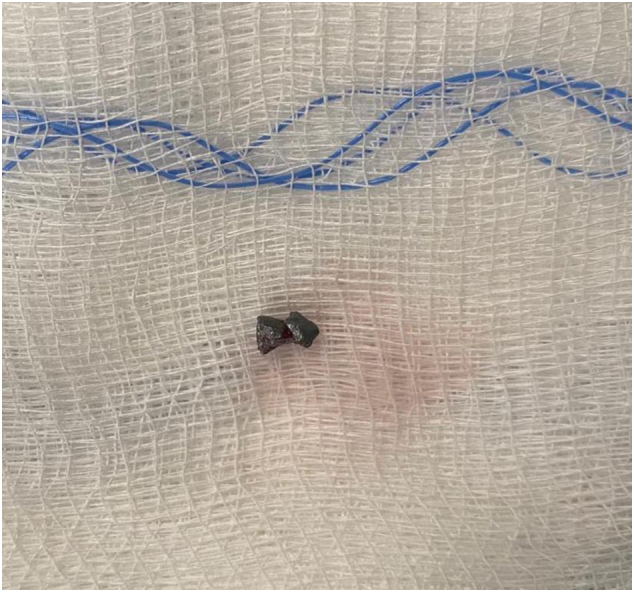
Foreign object after removal. Post-operative image showing the air gun pellet after removal from the frontal sinus.

The attending surgeon conducted a modified Lothrop procedure by drilling the frontal sinus floor from the left lamina papyracea to the contralateral lamina papyracea (Fig. 3). The pellet was identified and successfully removed (Fig. 4) (Video 1). The skull base defect seen in the preoperative scans was examined, and a cerebrospinal fluid (CSF) leak was identified. The defect was repaired using an on-lay mucosal graft. We followed up with the patient after 11 weeks in the outpatient clinic with no concerns.

## 3 Discussion

Foreign bodies rarely present in the paranasal sinuses, and most cases may present with a wide variety of symptoms [14]. Previously, foreign bodies in the paranasal sinuses may have gone unnoticed; however, with advancements in radiographic imaging, it is rare for foreign bodies to present in a late stage [14]. In one retrospective study, 15 % of retained metallic objects were found in the frontal sinus in head trauma patients [2]. The literature reports a variety of objects that have been retained in the frontal sinus, including glass [4], a silicone tube [15], and air gun pellets [12]. Reported side effects of retained foreign body in the paranasal sinuses include abscess formation, lead poisoning, and, in rare cases, meningitis [5].

Management is directed by multiple factors, including the patient's hemodynamic status, the site of the foreign body, and the degree of involvement of the surrounding structures [6], [7], [8]. Historically, these types of injuries were treated by conventional open surgeries. However, treatment with endoscopic approaches has become more feasible due to advances in technology, surgeons' skills, and understanding of the endoscopic sinonasal anatomy, which allows for successful, less invasive surgeries [16]. Moreover, it provides a cosmetic advantage due to a lesser chance of facial scarring [12]. Guidelines for retrieving foreign objects have not yet been set in stone; however, a proposed guideline suggests endoscopic intervention when possible if there is a risk of infection or persistent CSF leakage [2].

ESS is a minimally invasive technique that improves sinus ventilation and commonly treats chronic rhinosinusitis [17]. Reported side effects of ESS include infection, bleeding, CSF leakage, and, rarely, blindness [17]. Other than being minimally invasive, ESS presents many advantages, including decreased postoperative discomfort and risk of damage to the nerve supply of the teeth [10]. Extended sinus surgeries are used to create larger communication between the sinuses and the nasal cavity for various reasons, such as management of recalcitrant sinus disease and resection of sinonasal tumors. In addition, this approach will result in better exposure of the skull base that can facilitate the repair of a CSF leak [18]. In this case, the extended frontal sinus surgery through a modified Lothrop procedure (also known as Draf III procedure) helped visualize the defect and facilitated the repair while maintaining the frontal sinus drainage. Moreover, it provided safe entry to the right frontal sinus after identifying the posterior table through the healthy left frontal sinus that was entered first. Only then the floor of both frontal sinuses was drilled down while keeping the posterior table under direct visualization. This method ensured that the pellet was not accidentally dislodged intracranially through the defect in the right, which is a possible complication if a standard approach was used to enter the right frontal sinus through the frontal recess. A minor skull base defect measuring 0.5 × 0.5 cm with CSF leak was identified following removal of the pellet and was repaired using a mucosal on-lay graft. Eleven weeks postoperatively, the patient was doing well with no CSF leak or frontal sinus obstruction.

A similar case reported a 16-year-old male presenting with an air gun pellet in the frontal sinus. The pellet was successfully removed using an endoscopic procedure, and there was no evidence of CSF leakage [12]. The endoscopic approach used in this case was the Draf IIa approach, while our case used the modified Lothrop or Draf III approach.

## 4 Conclusion

To our knowledge, this is the only other reported case of ESS usage to retrieve a retained air gun pellet in the frontal sinus [12]. Based on our experience with this patient and similar literature, ESS should be considered a treatment option for patients with retained foreign objects in the frontal sinus.

The following is the supplementary data related to this article.

Video 1 This is an intraoperative video showing the different surgical steps taken to complete a modified Lothrop procedure to facilitate the removal of the foreign body from the right frontal sinus. Inspection of the posterior table of the frontal sinus afterwards revealed a skull base defect and CSF leak that was repaired using an on-lay free mucosal graft. The surgery was done using a 70° nasal endoscope. Video 1

## Provenance and peer review

Not commissioned, externally peer-reviewed.

## Availability of data and material

Not applicable.

## Code availability

Not applicable.

## Ethical approval

Patient anonymity is maintained throughout this manuscript, and consent was obtained for publication from the patient.

## Funding

This study did not receive funding from any source.

## Guarantor

Dr. Ghassan al Okby.

## Research registration number

N/A.

## CRediT authorship contribution statement

HYS, TZA, REE, BNS, drafted the manuscript GA contributed to reviewing and finalizing the manuscript. He also provided the imaging findings and their interpretation for the case presentation section. All authors reviewed the manuscript for intellectual content and approved the submission.

## Declaration of competing interest

The authors declare no conflict of interest.
